# The Misdiagnosis Tracker: Enhancing Diagnostic Reasoning Through Cognitive Bias Awareness and Error Analysis

**DOI:** 10.3390/jcm14124139

**Published:** 2025-06-11

**Authors:** Zeinab Mutlak, Noor Saqer, See Chai Carol Chan, Azeem Majeed, Waseem Jerjes

**Affiliations:** 1 Brighton and Sussex Medical School, University of Sussex, Brighton BN1 9PX, UK; z.mutlak1@uni.bsms.ac.uk (Z.M.); n.saqer1@uni.bsms.ac.uk (N.S.); 2 Faculty of Medicine, Imperial College London, 86 Wood Lane, London W12 0BZ, UK; carol.chan6@nhs.net (S.C.C.C.); a.majeed@imperial.ac.uk (A.M.)

**Keywords:** diagnostic reasoning, cognitive bias, medical education, misdiagnosis, reflective learning, atypical presentations

## Abstract

**Introduction:** Diagnostic accuracy is essential for good-quality medical practice, and yet diagnostic errors remain widespread, influencing patient outcomes, healthcare costs, and clinician confidence. “Misdiagnosis Tracker” aims to instil error analysis into medical education and shift the focus toward the learning of lessons from errors in diagnosis. This study investigates how systematic review of diagnostic errors enhances medical students’ competence in diagnostic reasoning, identification of cognitive bias, and identification of atypical presentations. **Methods:** A cohort of 65 final-year medical students participated in a structured three-phase educational intervention comprising preparation, case study analysis, and reflection. Students examined 20 diagnostic error case studies to identify contributory factors, such as cognitive biases, atypical presentations, and systemic barriers. Quantitative data were obtained from pre- and post-study surveys assessing confidence and knowledge, while qualitative insights were gathered through group reports, reflective journals, and debriefing sessions. **Results:** The results indicated significant improvements in confidence in diagnostic reasoning (3.0 to 4.4, *p* < 0.01), awareness of cognitive bias (2.9 to 4.5, *p* < 0.01), and recognition of atypical presentations (3.1 to 4.6, *p* < 0.01). Qualitative results highlighted increased insight into red flags, systemic issues, and reflective development. Actionable recommendations made by the students were systematic diagnostic frameworks, better interprofessional communication, and focused education on cognitive bias. **Conclusion:** This study illustrates how the “Misdiagnosis Tracker” greatly enhances medical students’ reflective and diagnostic reasoning skills and better prepares them for clinical practice. By fostering an environment of learning from diagnostic mistakes, this approach could ultimately result in fewer diagnostic errors and improved patient outcomes. Longitudinal studies are essential to determine the long-term effect of this model on clinical competence and its application in different education settings.

## 1. Introduction

Diagnostic accuracy is essential for successful medical practice, yet errors in diagnosis are an intractable problem with implications for patient outcomes, health expenditure, and clinician confidence [1]. Around 5% of outpatient consultations result in misdiagnosis, although this figure is considerably higher in patients with complex or atypical presentations [2]. These errors may arise due to a variety of reasons, including cognitive bias, atypical presentations, information gathering, and systemic issues, such as pressures of time and compartmentalised care [3,4].

For clarity, cognitive bias refers to systematic thinking errors that influence clinical judgment (e.g., anchoring or premature closure); atypical presentations denote symptoms or signs that deviate from textbook cases; and red flags are clinical warning signs suggestive of potentially serious underlying conditions.

Although diagnostic error is well recognised, medical education continues to remain inattentive to the process of diagnosis and seldom draws on these diagnostic failure cases as learning opportunities [4,5].

Misdiagnoses offer a unique window into the complexity of clinical reasoning and decision making [6]. Reviewing such misdiagnoses allows clinicians and trainees to identify common underlying patterns, refine differential diagnosis, and develop strategies to avoid future errors [7,8]. For instance, failure to diagnose myocardial infarction, appendicitis, or autoimmune disorders could be due to an atypical presentation of the disease or overreliance on anchoring heuristics [9,10,11]. Going through such cases can not only enable an opportunity to learn about the specific conditions but, more importantly, learn about systematic and cognitive factors influencing the accuracy of diagnosis [12].

This study, “The Misdiagnosis Tracker”, offers an educational strategy to equip medical students with the competence to critically analyse patterns of diagnostic errors. In a departure from a traditional teaching method that emphasises correct answers, the programme instead focuses on learning from mistakes [13]. Using both real-life and simulated case studies, learners will develop a greater awareness of diagnostic reasoning, red flag recognition, atypical presentations, and cognitive biases that contribute to errors in clinical reasoning [14,15].

The pilot study will assess the impact of this novel approach on students’ diagnostic reasoning, recognition of clinical pitfalls in practice, and confidence in managing uncertain and challenging cases. “The Misdiagnosis Tracker” aims to foster an ethos of error-based learning to enable future clinicians to approach diagnostic challenges with sharper acuity and greater resilience. Hence, the purpose of this study is to determine whether systematic review of diagnostic errors improves medical students’ diagnostic reasoning, recognition of cognitive bias, and identification of red flags in atypical presentations.

While prior studies have examined elements of diagnostic reasoning or cognitive bias in isolation, few have operationalised a structured educational intervention that combines real case error analysis with reflective practice in a way that is scalable and reproducible. This study contributes to a growing body of literature calling for diagnostic error to be embedded into the formal curriculum, and it offers a model that bridges theoretical understanding with experiential learning.

## 2. Methods

### 2.1. Study Design

This observational cohort study employed a systematic three-phase educational intervention to improve medical students’ diagnostic reasoning. A pre- and post-intervention design assessed changes in confidence, recognition of cognitive bias, and identification of atypical presentations (Appendix A). The intervention involved real case discussions, small-group analyses, and reflective exercises. Quantitative data were gathered from surveys, while qualitative data came from group reports, reflective journals, and debriefing sessions.

While this study did not include a control group, the pre–post design is a widely accepted approach in educational evaluations, particularly within service improvement contexts. Control groups were not feasible in this setting due to curriculum-wide implementation and ethical considerations around withholding educational interventions shown to be beneficial. Nonetheless, this design allows for valuable insight into individual learning progression and offers a pragmatic model for assessing impact in real-world academic environments.

### 2.2. Intervention Phases

The intervention had three structured phases: preparation, case study analysis, and review/reflection. Table 1 outlines the structure, activities, and intended learning outcomes of each phase.

During the preparation phase, students attended a workshop introducing misdiagnosis, major error statistics, and common conditions at risk of misdiagnosis. Cognitive biases, such as anchoring, availability, and confirmation bias, were explored, building on frameworks described by Croskerry [16] and Graber et al. [17], who have extensively documented the role of cognitive error in diagnostic practice. A systematic approach to identifying misdiagnosis patterns was introduced. Students completed a pre-study survey to establish baseline confidence and awareness levels.

The second phase focused on case study analysis. Students examined twenty real and simulated cases illustrating different misdiagnosis patterns, including atypical presentations, cognitive biases, and systemic factors like poor communication. Small teams of students systematically analysed cases to identify errors in history-taking, examination, and test interpretation. Teams debated different cognitive and systemic causes, red flags, and opportunities for correction, documenting their analyses in formal reports, which were later discussed in structured debriefing sessions. A detailed breakdown of the 20 diagnostic error case studies analysed by students—including primary diagnosis, misdiagnosis, contributing biases, and proposed solutions—is presented in Table 2

In the final stage, students participated in debriefing sessions with clinical educators to present findings and discuss patterns across cases. They reflected on their evolving reasoning, cognitive bias awareness, and strategies for reducing future diagnostic errors. A post-study survey measured changes in confidence, knowledge, and application of diagnostic principles. Reflective journals captured insights on how the intervention influenced clinical reasoning and ability to detect atypical presentations.

The diagnostic cases used in the intervention were selected and developed by a panel of three senior clinical educators with expertise in diagnostic reasoning and medical education. Case content was drawn from real patient incidents anonymised for teaching purposes and supplemented with fictionalised scenarios designed to reflect diagnostic uncertainty, cognitive bias, and atypical presentations. Each case was reviewed for plausibility, learning value, and complexity and standardised using a common template that included presenting features, contextual cues, red herrings, and outcome summaries. This standardisation ensured consistency across cases and alignment with intended learning objectives.

### 2.3. Setting

The study involved final-year students from three London medical schools who had undertaken primary care placements. Sessions were held in-person and remotely to accommodate schedules. The structured intervention lasted six weeks, incorporating formal case analysis, small-group discussions, and debriefing workshops led by clinical educators. Data collection took place between February 2023 and October 2024 in controlled academic settings.

### 2.4. Participants

Sixty-five final-year medical students were recruited via direct email based on interest in diagnostic reasoning. Inclusion criteria required at least two completed clinical rotations across different specialties to ensure meaningful case analysis engagement. Participants had prior exposure to specialties including internal medicine, surgery, paediatrics, and primary care. Students who had previously completed formal coursework or workshops specifically focused on diagnostic error analysis were excluded to ensure a consistent baseline of prior exposure. All participants provided written consent. Recruitment aimed for a representative sample across different medical schools. No students were lost to follow-up.

### 2.5. Variables

Variables were categorised as independent, dependent, and potential confounders. The independent variable was the structured three-phase educational intervention. The dependent variables included changes in diagnostic confidence, cognitive bias awareness, and recognition of atypical presentations, assessed via pre- and post-study surveys. Qualitative data from reflective journals and group reports were used to explore shifts in diagnostic reasoning. Potential confounders included prior clinical exposure, curriculum-based discussions of diagnostic errors, and awareness of cognitive bias. All participants received identical educational material.

### 2.6. Data Sources and Measurement

Data were collected from multiple sources. Pre- and post-surveys using 5-point Likert-scale items measured confidence, bias awareness, and ability to identify atypical presentations. The survey instruments were developed by the authors, drawing on validated frameworks from prior studies in diagnostic reasoning and cognitive bias [16,17]. Group reports documented case discussions, diagnostic pitfalls, and strategies for error prevention. Reflective journals captured insights on diagnostic error recognition and bias minimisation. Facilitated debriefing sessions reinforced learning and highlighted key takeaways. Standardised frameworks ensured consistent data collection. Surveys were anonymised to encourage honest responses. Data were securely stored and accessible only to the research team.

### 2.7. Bias Control

Multiple strategies were implemented to reduce potential sources of bias, following recommended practices in educational and health research [18]. Selection bias was minimised by recruiting from multiple institutions using consistent criteria. Response and social desirability bias were addressed through anonymous surveys and reassurances regarding academic neutrality. Recall bias was reduced by timing surveys immediately before and after the intervention. Observer and confirmation bias were mitigated by having multiple independent researchers analyse qualitative data using structured thematic frameworks that promoted critical reflection and challenged initial assumptions.

### 2.8. Study Size

Sample size was determined through power analysis to detect significant changes in diagnostic reasoning and bias awareness. With an expected effect size of 0.6, a minimum of 52 participants was required for 80% power at a significance level of 0.05. Accounting for attrition, 65 students were enrolled. The structured intervention minimised variability and enhanced result reliability.

The sample size was calculated using G*Power version 3.1 [19] based on a two-tailed paired t-test, with an expected medium effect size derived from prior studies on diagnostic reasoning interventions [17], an alpha of 0.05, and power of 0.8.

### 2.9. Quantitative Variables

A formal thematic analysis was undertaken using Braun and Clarke’s six-step framework (familiarisation, coding, theme development, review, definition, and writing up). Two researchers independently coded the reflective journals using an inductive approach to identify emergent themes. Coding discrepancies were discussed and resolved through consensus, and NVivo software (version 14) was used to assist with data management and theme organisation.

Pre- and post-intervention surveys measured confidence in diagnostic reasoning, bias awareness, and recognition of atypical presentations. Confidence was assessed based on the ability to formulate differential diagnoses and avoid premature closure. Bias awareness was evaluated through students’ ability to recognise and mitigate anchoring, availability, and premature closure biases. Recognition of atypical presentations was assessed based on students’ ability to identify non-classical symptoms in commonly misdiagnosed conditions. Secondary outcomes included the perceived ability to recognise red flags and the perceived influence of systemic factors on diagnosis. Scores were analysed using means and standard deviations, with paired t-tests assessing statistical significance.

### 2.10. Statistical Methods

Statistical analyses evaluated the intervention’s impact on diagnostic reasoning, bias awareness, and recognition of atypical presentations. Paired t-tests were conducted to compare pre- and post-study survey scores. The Shapiro–Wilk test was used to assess data normality. The Wilcoxon signed-rank test was used for non-normally distributed data. Effect size was calculated using Cohen’s d to estimate the magnitude of change. Thematic analysis of qualitative data was conducted independently by two analysts to identify patterns, with interrater reliability measured using Cohen’s kappa. Sensitivity analyses were used to examine subgroup differences. Data analysis was conducted using SPSS (version 27.0), adhering to educational research guidelines.

### 2.11. Ethical Considerations

This study was classified as an educational evaluation and did not require formal ethics approval, in accordance with institutional guidelines. Written informed consent was obtained, participation was voluntary and anonymous, and students could withdraw at any time. Data collection adhered to General Data Protection Regulation (GDPR) regulations, with all identifiers removed prior to analysis.

## 3. Results

### 3.1. Participants

A total of 65 final-year medical students participated in the study and completed both the pre- and post-intervention assessments, resulting in a 100% retention rate. The participants were recruited from three medical schools in London, ensuring diversity in clinical experiences. The cohort comprised 38 female students (58%) and 27 male students (42%), with a mean age of 24.3 ± 1.2 years.

Previous clinical exposure of the students was varied, with all having had internal medicine, surgery, primary care, and paediatrics rotations. Fewer had exposure to emergency medicine (40 students) and radiology (12 students). Baseline survey findings indicated that 49 had no previous formal teaching in diagnostic error and 58 had no formal teaching in cognitive bias. Baseline confidence in diagnostic reasoning was low in most instances, with 41 of the students below the mid-point on the Likert scale and only 11 regarding themselves as confident or very confident. Table 3 summarises the quantitative findings, showing pre- and post-study scores and highlighting areas of significant improvement.

All students participated in the formalised case discussions and reflective exercises and added to the qualitative data set. No students withdrew from the study, and all submitted the required group reports and post-intervention reflections.

### 3.2. Descriptive Data

Baseline assessments revealed variability in students’ prior exposure to and confidence in diagnostic reasoning and cognitive bias. Mean confidence in diagnostic reasoning was 3.0 ± 0.9, and 41 of the students had confidence below the midline (Table 3). Awareness of cognitive bias was low at a mean of 2.9 ± 0.8, and only 16 of the students knew how cognitive bias results in diagnostic errors.

Similarly, recognition of non-classical presentations at baseline was low at 3.1 ± 0.7, and 38 students acknowledged difficulty in recognising atypical symptoms of commonly misdiagnosed conditions. Before the study, 49 students had no formal teaching in errors in diagnosis, and 58 had no formal teaching in cognitive bias. However, 57 students believed errors in diagnosis were a significant issue in practice, and 61 believed formal teaching in misdiagnosis analysis would be beneficial.

At baseline, recognition of red flags in complex clinical cases varied, with a mean score of 2.8 ± 0.9, which reflected a general lack of confidence. Similarly, recognition of systemic barriers to accurate diagnosis, such as pressures of time and fragmented care, was moderate (mean score of 3.2 ± 0.6).

### 3.3. Outcome Data

**Figure 1:**
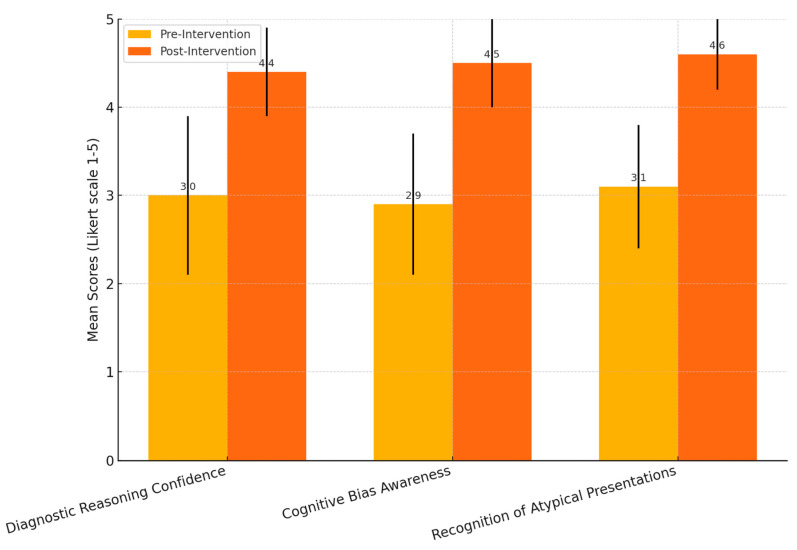
Comparison of mean pre- and post-intervention scores (Likert scale: 1 = lowest, 5 = highest) for diagnostic reasoning confidence, cognitive bias awareness, and recognition of atypical presentations. Error bars represent standard deviations, demonstrating significant increases across all categories after the educational intervention.

Post-intervention surveys demonstrated significant improvement in all measured domains. Confidence in recognising cognitive bias increased from 2.9 ± 0.8 to 4.5 ± 0.5 (*p* < 0.01). Ability to recognise atypical presentations increased from 3.1 ± 0.7 to 4.6 ± 0.4 (*p* < 0.01). Recognition of systemic causes of misdiagnosis increased from 3.2 ± 0.6 to 4.7 ± 0.5 (*p* < 0.01). Diagnostic reasoning confidence increased from 3.0 ± 0.9 to 4.4 ± 0.5 (*p* < 0.01). These findings support the value of systematic error analysis in improving students’ diagnostic reasoning, bias awareness, and recognition of red flags in challenging cases. The calculated effect sizes (Cohen’s d) were large across all measured domains—ranging from 2.06 to 2.63—demonstrating strong educational impact beyond statistical significance. Figure 1 shows the significant shift in students’ confidence and awareness across all measured domains.

### 3.4. Main Results

Analysis of all 20 diagnostic error case studies revealed recurrent patterns. Half of the instances were atypical presentations and instances of missed diagnoses. For example, myocardial infarction was misdiagnosed as gastritis due to an initial gastrointestinal presentation. In another case, multiple sclerosis was diagnosed as musculoskeletal trauma as students did not recognise minor neurological signs.

Cognitive errors contributed to 65% of the misdiagnoses, with anchoring and premature closure being most common. In one case, presentation of progressive weakness was misdiagnosed as musculoskeletal strain, leading the students to anchor on this and exclude neurological disorders, such as Guillain–Barré syndrome. Confirmation bias occurred in a case of chronic cough and weight loss, where students presumed recurring respiratory infection and delayed identifying the diagnosis of lung cancer.

**Figure 2:**
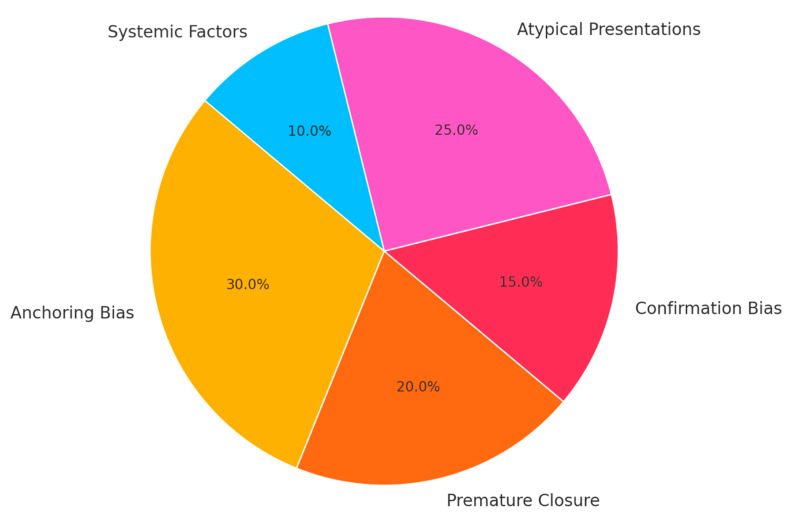
Pie chart illustrating the proportional distribution of diagnostic errors identified through analysis of 20 diagnostic error case studies. Cognitive biases (anchoring, premature closure, confirmation bias), atypical presentations, and systemic factors represent the main categories of error contributing to misdiagnosis in the study cohort.

Systemic issues accounted for 40% of the cases, most commonly involving communication breakdowns, time pressures, and lack of follow-up protocols. One of the cases involved a delayed diagnosis of colorectal cancer due to failure to act on an abnormal screening test result, which emphasised the need for follow-up protocols. As illustrated in Figure 2, cognitive biases, atypical presentations, and systemic factors were the predominant categories of diagnostic error identified. By the final case discussion session, 95% of the students could explain how cognitive bias, systemic inefficiencies, and clinical complexity come together in generating diagnostic errors and demonstrated improved critical thinking in diagnostic reasoning.

### 3.5. Other Analyses

Thematic analysis of reflective journals provided further insight into the impact of this intervention on students’ clinical thinking. Students highlighted the importance of stepping back to re-assess clinical situations and being aware of thought traps. One student said the exercise revealed how easily first impressions could lead to information being overlooked and reinforced the importance of re-examining cases again. Another student reflected that they had previously not considered how non-specific symptoms, such as fatigue, could be a sign of a life-threatening underlying illness. As a result of the exercise, students reported a greater appreciation for differential diagnoses and careful clinical assessment. Themes from students’ reflective journals were analysed and are presented in Table 4, along with illustrative quotations and implications.

**Figure 3:**
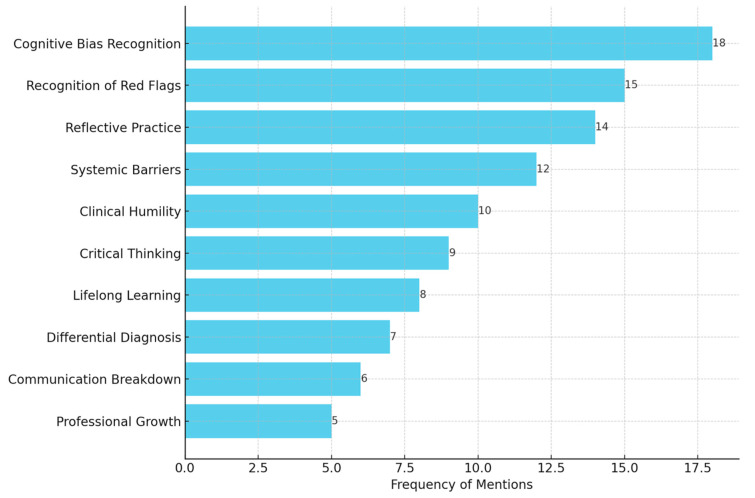
Horizontal bar graph illustrating the relative frequency with which key qualitative themes were identified in students’ reflective journals. Higher frequency indicates themes that students emphasised most strongly as critical insights gained from analysing diagnostic errors during the educational intervention.

Students also acknowledged the importance of humility in clinical practice and that errors in diagnosis are unavoidable but could be used as learning points. One participant described this intervention as an opportunity to embrace and learn from errors to enhance their ability to diagnose. Most students indicated that the systematic examination of errors in diagnosis had a profound effect on their clinical reasoning process. The distribution of key themes across reflective journals is shown in Figure 3, highlighting the prominence of bias recognition and red flag awareness.

### 3.6. Common Patterns and Proposed Solutions

Group reports identified dominant patterns of diagnostic errors and proposed solutions. Anchoring and premature closure were the most frequent cognitive errors, contributing to 65% of misdiagnoses by causing fixation on an early diagnosis and failure to consider other alternatives. Red flags were overlooked or dismissed in 58% of cases, typically due to inadequate re-evaluation of symptoms when new information became available. Systemic barriers, such as fragmented care and absence of follow-up protocols, impacted 40% of misdiagnoses.

Students proposed various solutions to reduce diagnostic errors, including periodic differential diagnosis review to prevent cognitive fixation, improved communication between departments to enhance care coordination, and utilisation of systematic checklists to prevent overlooking red flags.

## 4. Discussion

### 4.1. Key Results

This study demonstrates the potential of diagnostic error analysis in improving medical students’ diagnostic reasoning and decision making. By examining misdiagnosis cases, students became more aware of cognitive bias, atypical presentations, and systemic barriers to accurate diagnosis. This intervention led to significant improvement in confidence, awareness, and ability to handle complex clinical cases in favour of the use of diagnostic error analysis in medical education.

Pre-intervention answers revealed limited student knowledge and minimal experience in recognising cognitive biases and their impact on clinical reasoning. Following the intervention, students showed improved capacity to identify and manage such biases, as reflected in improved confidence scores. These findings suggest that structured learning of misdiagnosis enables students to recognise and avoid common errors in clinical practice [20].

Analysis of case studies revealed consistent patterns of diagnostic errors. Anchoring and premature closure were established as significant contributors in 65% of instances. Students reflected on how early diagnostic impressions impacted their judgments and led them away from considering other explanations despite having additional information. This finding aligns with the previous literature, such as the work of Graber et al. [17] and Blumenthal-Barby and Krieger [20], who identify cognitive bias—particularly anchoring and premature closure—as a critical contributor to diagnostic delay and error.

One of the major issues discussed was the importance of identifying atypical presentations. Conditions like autoimmune disease and myocardial infarction can have atypical symptoms, therefore increasing the risk of misdiagnosis [21,22]. Students’ reflections demonstrated growing awareness and appreciation for maintaining broad differential diagnosis, especially when patients’ symptoms deviate from the classic descriptions in textbooks [23,24,25].

Systemic failures, including communication breakdowns, time pressures, and lack of follow-up, were also identified as root causes of diagnostic errors. In another scenario, delayed diagnosis of colorectal cancer resulted from the failure to follow up on abnormal test results and reflects an organisational failure in perpetuating the error in diagnosis. Both diligence at the individual level and systemic reform in health facilities will be necessary in order to address such errors and facilitate timely and accurate diagnoses [26].

### 4.2. Interpretation

Diagnostic errors remain under-researched in traditional medical education [9,12,19]. Most programmes place a priority on reinforcing correct diagnoses rather than actively exploring errors in diagnosis and consequently overlook valuable learning opportunities [18,27]. The results of this study show that systematic examination of errors in diagnosis can improve the diagnostic reasoning of students by helping them critically review cases, test their assumptions, and enhance clinical judgment. “Misdiagnosis Tracker” is an innovation in medical education to shift from a conventional model focusing on reaching accurate diagnoses to a model with an emphasis on learning from errors.

Traditional medical education often emphasises knowledge acquisition and correct answers, typically through structured tutorials, MCQs, and protocol-based simulations [2,8,20]. While these strategies build foundational knowledge, they seldom address the root causes of diagnostic errors. The “Misdiagnosis Tracker” differs by encouraging students to explore diagnostic failures, cognitive pitfalls, and system-level barriers through structured reflection. This comparison highlights the added value of incorporating metacognitive training into clinical education. To integrate this model into broader curricula, medical schools could embed diagnostic error analysis into early clinical exposure modules, simulation-based assessments, or interprofessional learning sessions. Longitudinal reinforcement through reflective portfolios and quality improvement exercises may support a sustained impact on diagnostic safety.

It promotes students’ conceptualisation of clinical reasoning and prepares them better to cope with uncertainty in diagnosis, particularly in challenging cases [12,19].

The most significant improvement in this study was students’ recognition of cognitive biases and red flags. Through case discussions, students learned systematic approaches to managing challenging cases and developed reflective and critical thinking. These are essential competences in managing uncertainty in diagnosis, particularly in low-resource and/or high-risk clinical settings [20,28].

While the increase of 1.4 to 1.6 points on a 5-point Likert scale may appear modest, such gains are educationally meaningful. A shift from “neutral” to “agree” or from “disagree” to “agree” often reflects a substantive change in mindset and preparedness. In clinical contexts, this could translate into improved recognition of diagnostic red flags, reduced reliance on heuristics, and greater willingness to question premature conclusions, especially in ambiguous cases. These behavioural shifts are crucial in enhancing diagnostic accuracy and minimising preventable errors, particularly in high-pressure or uncertain clinical environments.

These findings also show the value of incorporating reflective exercises into medical curricula. Analysis of students’ reflective journals demonstrated a significant shift in their process of diagnostic reasoning. Many of the students described how the exercise profoundly shifted their approach to the clinic and highlighted the importance of questioning assumptions and being open to differential diagnoses. Such findings highlight the advantages of building a culture of humility and continuous learning where mistakes are viewed as an opportunity for growth and not failure [28,29].

### 4.3. Limitations

There are several limitations to this study. The small number of participants (65 students) limits the study’s generalisability. Even though the intervention produced statistically significant gains, larger-scale studies are needed to determine whether such gains result in permanent changes in clinical practice. A second issue is the use of simulated case studies; as useful as they are in an educational setting, they lack the subtlety of real-case diagnostic dilemmas.

While all cases were developed and reviewed by experienced clinical educators to ensure plausibility and relevance, we acknowledge that simulated diagnostic error cases may still lack full external validity, and learners’ responses could be influenced by how realistic or typical they perceive the cases to be.

Future studies need to explore how skills are implemented in practice in the clinical setting in order to test the effectiveness of the intervention in real life.

Another limitation is the absence of a control group, which restricts our ability to attribute observed improvements solely to the intervention. Although this pre–post design is widely accepted in service evaluation and educational research, we recognise that other factors, such as concurrent clinical placements, informal learning, or natural progression in confidence, may have contributed to the observed gains. Control groups were not employed in this pilot due to ethical concerns around denying a promising educational intervention and logistical challenges in creating isolated study groups within an integrated curriculum. However, future research could consider quasi-experimental or stepped-wedge designs to more directly assess causality and control for external influences.

Other potential confounding variables, such as prior teaching in cognitive bias, prior exposure to diagnostic error cases, or variability in individual clinical placements, were not formally controlled for, and their influence cannot be ruled out. However, baseline data collected as part of the pre-intervention survey indicated that 58 out of 65 students had no prior formal teaching in cognitive bias, and 49 had no exposure to diagnostic error analysis, suggesting a relatively uniform starting point for most participants.

Future studies could adopt a controlled or randomised design to isolate the specific impact of the intervention. Additionally, incorporating objective performance measures, such as performance in simulated diagnostic scenarios or clinical evaluations, would provide a more robust assessment of diagnostic competence beyond self-reported confidence.

Furthermore, the research focused on the opinions of the students and did not elicit any direct feedback from patients and healthcare professionals. Future iterations of this intervention would be improved by incorporating opinions from pharmacists, nurses, and patient advocates to bring richer insight into the process of diagnosis and systemic issues underlying errors.

### 4.4. Generalisability

Despite its limitations, this study provides valuable information on the potential benefits of implementing diagnostic error analysis in medical education. While the sample size was 65 students, these students were drawn from three separate medical schools in London, providing a degree of educational diversity. Moreover, the sample size was not arbitrary; a power analysis using G*Power (Version 3.1) indicated that a minimum of 52 participants was required to detect a medium effect size (0.6) at 80% power and alpha 0.05. This study exceeded that threshold, enhancing the robustness of our conclusions.

We recognise, however, that expanding the participant pool across multiple regions and institutions, as well as including different specialties, would increase generalisability. Future research will explore implementation across wider educational settings to evaluate whether similar outcomes are observed and how local factors might influence results.

The systematic process implemented in this intervention could be adapted in other medical schools and health systems. For example, including systematic frameworks for error analysis in medical curricula enables students to identify and address sources of error in an orderly manner. Checklists and decision aids have the ability to cause clinicians to reconsider their differential diagnoses and overcome biases in cognition [2,29]. Findings concur with growing evidence that instruction in diagnostic reasoning to improve clinical judgment should emphasise not only accurate diagnosis but also reflective learning from mistakes.

These initial findings are promising, but to assess durability and practical value, longitudinal research is needed to examine whether the gains in diagnostic reasoning and bias awareness are sustained in postgraduate settings. Future studies should explore whether improved reasoning skills translate into measurable reductions in diagnostic errors, improved patient outcomes, and enhanced clinical confidence over time.

Secondly, further emphasis on cognitive biases and atypical presentations in medical education is needed [3,27,30]. This study demonstrates the manner in which intense debate on such topics significantly enhances the capacity of students to recognise and overcome biases. Expanding such debate into real life and cross-disciplinary perspectives could further optimise learning [5,12,31,32].

In particular, incorporating interprofessional voices, such as pharmacists, nurses, and patient advocates, into both educational delivery and research design could enhance understanding of systemic contributors to diagnostic error and support a more holistic approach to diagnostic safety.

Thirdly, systemic barriers have to be tackled through collaboration between health and education organisations. All students consistently mentioned poor communication and lack of follow-up as major causes of diagnostic errors. Interprofessional practice and systems-based practice modules could enhance the readiness of future clinicians to handle such problems [33,34,35].

Longitudinal studies will be needed to evaluate the durability of these gains, including whether students maintain these skills during foundational training and apply them effectively in real-world diagnostic challenges. Future work could link educational interventions to clinical outcomes, such as diagnostic error rates, near-miss reporting, or supervisor evaluations, to assess long-term impact.

Larger and more representative samples also need to be investigated to ascertain the extent to which variations in education settings and different cultural contexts influence the effectiveness of error analysis in diagnosis.

Medical curricula can integrate diagnostic error analysis through structured debriefing sessions following clinical placements, simulation-based learning, and small-group tutorials focused on cognitive bias reflection. Embedding these components longitudinally across multiple years of training reinforces reflective habits.

To ensure sustainability, this intervention could be embedded into existing clinical placements or integrated into reflective portfolio requirements across medical training years. Faculty development programs can also help train clinical educators to deliver error analysis sessions consistently and effectively. Involving interprofessional teams, such as nurses, pharmacists, patient safety officers, and patient advocates, can enrich feedback and provide a broader, systems-based perspective on diagnostic error. Looking ahead, longitudinal studies are needed to assess whether improved diagnostic reasoning during training translates into measurable patient safety outcomes.

## 5. Conclusions

This work provides clear evidence in favour of systematic error analysis as a method for improving clinical reasoning and preparing medical trainees for the diagnosis-related issues they will have to deal with in clinical practice. The implications of the study are that an integration of cognitive bias education, reflective learning, and systems-based approaches into medical education can enhance patient safety and diagnostic accuracy and result in better health outcomes.

## Figures and Tables

**Table 1:** Overview of diagnostic error analysis framework.

Phase	Key Activities	Objectives	Outcomes Measured
Phase 1: Preparation	- Introductory workshop on diagnostic errors and cognitive biases. - Overview of common misdiagnosis patterns and systemic factors. - Pre-study survey to assess baseline confidence and familiarity with diagnostic reasoning challenges.	- Establish foundational knowledge of diagnostic errors and contributing factors. - Introduce structured frameworks for case analysis.	- Baseline confidence and knowledge scores. - Initial attitudes toward the importance of learning from misdiagnoses.
Phase 2: Case study analysis	- Analysis of 20 diagnostic error case studies representing atypical presentations, cognitive biases, and systemic barriers. - Group discussions to identify error patterns, missed red flags, and contributing factors. - Documentation of findings in group reports.	- Develop skills to systematically analyse diagnostic errors. - Enhance recognition of cognitive biases and atypical presentations. - Propose solutions to prevent diagnostic errors.	- Identification of common patterns in diagnostic errors. - Recognition of red flags and contributing factors. - Proposed strategies for error prevention.
Phase 3: Reflection and evaluation	- Facilitated debriefing sessions to discuss insights and share findings. - Post-study survey to assess changes in confidence and knowledge. - Submission of reflective journals documenting individual learning experiences.	- Encourage synthesis of learning from case studies. - Foster self-awareness of diagnostic reasoning strengths and weaknesses. - Identify actionable strategies for future practice.	- Post-study confidence and knowledge scores. - Thematic insights from reflective journals. - Strategies to mitigate diagnostic errors in clinical practice.

**Table 2:** Analysis of diagnostic errors—patterns, biases, and learning points.

Case	Diagnosis	Misdiagnosis	Bias Identified	Key Learning Point
1	Acute myocardial infarction	Gastritis	Anchoring	Consider cardiac causes in epigastric pain.
2	Stroke	Vertigo	Availability	Red flags in isolated dizziness.
3	Sepsis	Viral infection	Premature closure	Do not close diagnosis early.
4	Pulmonary embolism (PE)	Anxiety	Attribution	PE in anxious patients with tachycardia.
5	Appendicitis	Gastroenteritis	Confirmation	Recognise atypical signs of appendicitis.
6	Ectopic pregnancy	Urinary tract infection	Representativeness	Test pregnancy in women with abdominal pain.
7	Meningitis	Migraine	Anchoring	Lumbar puncture in suspected meningitis.
8	Lung cancer	Pneumonia	Overconfidence	Follow-up for unresolved symptoms.
9	Diabetic ketoacidosis	Hyperventilation	Attribution	Check metabolic causes in tachypnoea.
10	Subarachnoid haemorrhage	Tension headache	Failure to escalate	Image sudden severe headaches.
11	Ovarian torsion	Dysmenorrhea	Misattribution	Consider torsion in pelvic pain.
12	Celiac disease	Irritable bowel syndrome	Overgeneralisation	Screen for celiac in persistent gastrointestinal (GI) symptoms.
13	Renal colic	Musculoskeletal back pain	Context	Imaging for renal causes in young adults.
14	Aortic dissection	Acute coronary syndrome	Overlap	Dissection signs in chest pain.
15	Tuberculosis	Pneumonia	Failure to consider endemic causes	Endemic causes in immigrants.
16	Endocarditis	Viral infection	Anchoring	Echo in unexplained fevers.
17	Multiple sclerosis	Anxiety	Attribution	Magnetic resonance imaging for unexplained neuro signs.
18	Gallbladder perforation	Gastritis	Anchoring	Urgency in abdominal emergencies.
19	Hypercalcemia	Osteoporosis	Representativeness	Metabolic screen for chronic symptoms.
20	Pericarditis	Myocarditis	Overlap	Cardiac testing in chest pain.

**Table 3:** Quantitative results—improvements in diagnostic skills and confidence.

Metric	Pre-Study Score (Mean ± SD)	Post-Study Score (Mean ± SD)	Change	Cohen’s d	Key Observations
Confidence in identifying cognitive biases	2.9 ± 0.8	4.5 ± 0.5	+1.6 points (*p* < 0.01)	2.4	Students demonstrated a marked improvement in recognising and understanding biases like anchoring, availability, and confirmation bias.
Understanding of atypical presentations	3.1 ± 0.7	4.6 ± 0.4	+1.5 points (*p* < 0.01)	2.63	Increased awareness of non-classical symptoms in conditions like myocardial infarction, stroke, and autoimmune diseases.
Awareness of systemic factors	3.2 ± 0.6	4.7 ± 0.5	+1.5 points (*p* < 0.01)	2.72	Students better recognised the role of communication breakdowns, time constraints, and inadequate follow-ups in diagnostic errors.
Confidence in recognising red flags	2.8 ± 0.9	4.4 ± 0.5	+1.6 points (*p* < 0.01)	2.2	Improved ability to identify critical warning signs that may indicate serious conditions.
Overall diagnostic reasoning confidence	3.0 ± 0.9	4.4 ± 0.5	+1.4 points (*p* < 0.01)	1.92	Students reported increased confidence in approaching complex cases and navigating uncertainty in clinical scenarios.
Students confident in managing diagnostic errors (%)	40%	89%	+49%	-	The percentage of students feeling equipped to manage diagnostic challenges nearly doubled.
Recognition of the importance of learning from errors (%)	65%	97%	+32%	-	Students reported a stronger appreciation for analysing diagnostic errors as a tool for professional growth.
Students who recognised patterns of diagnostic errors (%)	48%	93%	+45%	-	The ability to systematically identify patterns, such as cognitive biases and systemic factors, improved significantly.

**Table 4:** Thematic analysis of reflective insights.

Theme	Description	Illustrative Student Quotes	Key Implications
Cognitive bias awareness	Students identified biases, such as anchoring, premature closure, and availability, as key contributors to misdiagnoses.	“I realised how often I rely on initial impressions and fail to reconsider the diagnosis when new information arises.”	Emphasises the need for deliberate re-evaluation of differential diagnoses throughout the diagnostic process.
Recognition of atypical presentations	Participants acknowledged the impact of non-classical symptoms in obscuring diagnoses and the importance of maintaining a broad differential.	“The patient with GI symptoms and a heart attack completely changed how I think about chest pain presentations.”	Highlights the necessity of training students to recognise red flags even when symptoms deviate from standard patterns.
Systemic barriers and oversight	Students identified systemic issues, such as inadequate follow-ups, time constraints, and poor communication, between healthcare providers.	“The missed test results in the cancer case really showed how systemic gaps can have devastating consequences.”	Stresses the importance of addressing organisational inefficiencies to improve diagnostic outcomes.
Red flags and missed opportunities	Students reported a deeper understanding of the importance of red flags and how their absence or dismissal contributed to misdiagnoses.	“I didn’t realise how often subtle signs are overlooked because they don’t fit the expected pattern.”	Reinforces the need for structured checklists and protocols to ensure red flags are not missed.
Emotional and reflective growth	Participants reflected on how the exercise fostered humility, resilience, and a greater awareness of their cognitive tendencies.	“This made me more comfortable with the idea that I will make mistakes, but I can learn from them to improve patient care.”	Encourages medical education programs to normalise discussions of errors as part of professional growth and continuous improvement.
Strategies for improvement	Students proposed actionable solutions, such as incorporating regular case reviews, enhancing communication systems, and using diagnostic decision aids.	“Using structured frameworks for case reviews could make a huge difference in preventing similar mistakes.”	Suggests that healthcare systems should adopt targeted interventions to support clinicians in reducing diagnostic errors.
Importance of lifelong learning	Students acknowledged the role of ongoing education and reflection in maintaining diagnostic proficiency and adapting to evolving challenges.	“This exercise taught me that learning doesn’t stop after med school—every mistake is a chance to grow.”	Underscores the value of embedding reflective practices and error analysis into continuous professional development initiatives.

## Data Availability

The datasets generated and/or analysed during the current quality improvement project are not publicly available due ethical reasons but are available from the corresponding author (W.J.) upon reasonable request.

## Appendix A

### Appendix A.1. Pre-Study Survey

Objective: To assess students’ baseline knowledge, confidence, and attitudes toward diagnostic reasoning and identifying cognitive biases.

Instructions: Please rate the following statements on a scale of 1 to 5, where: 1 = Strongly Disagree, 2 = Disagree, 3 = Neutral, 4 = Agree, 5 = Strongly Agree.

**Statement**
I feel confident in my ability to make accurate clinical diagnoses.
I understand the concept of cognitive biases and their impact on diagnostic accuracy.
I am familiar with red flags and atypical presentations of common conditions.
I feel confident distinguishing between disease progression and adverse drug effects.
I feel prepared to analyse diagnostic errors to improve my clinical reasoning skills.

Open-Ended Questions:

1. What do you hope to gain from this study on diagnostic reasoning?
2. What do you find most challenging about recognising and addressing diagnostic errors?
3. How do you currently address uncertainty in your diagnostic process?

### Appendix A.2. Post-Study Survey

Objective: To evaluate changes in students’ knowledge, confidence, and attitudes after completing the diagnostic reasoning program.

Instructions: Please rate the following statements on a scale of 1 to 5, where: 1 = Strongly Disagree, 2 = Disagree, 3 = Neutral, 4 = Agree, 5 = Strongly Agree.

**Statement**
I feel more confident in my ability to make accurate clinical diagnoses.
I can identify cognitive biases and their impact on diagnostic decision-making.
I am better able to recognise red flags and atypical presentations of conditions.
I feel more prepared to consider diagnostic errors in my clinical reasoning process.
This study has improved my ability to reflect on and learn from diagnostic errors.

Open-Ended Questions:

1. What was the most valuable insight you gained from analysing diagnostic errors?
2. How has this study changed your approach to diagnostic reasoning?
3. What strategies will you use to minimise diagnostic errors in your future practice?

### Appendix A.3. Reflective Journal Prompts

Objective: To gather qualitative insights into students’ learning experiences during the program.

1. Describe a specific case study that challenged your diagnostic reasoning. What did you learn from it?
2. Reflect on how cognitive biases influenced the diagnostic errors in the cases you analysed.
3. What strategies did you find most effective in identifying and addressing diagnostic errors?
4. How has this program influenced your understanding of atypical presentations and diagnostic red flags?
5. What changes do you plan to make in your clinical practice to minimise diagnostic errors?

### Appendix A.4. Group Analysis Report Template

Objective: To capture collaborative insights from student teams analysing case studies.

1. Summarise the key diagnostic error(s) identified in the case.
2. Discuss the cognitive bias(es) involved and how they influenced the outcome.
3. Propose specific strategies to prevent similar errors in future clinical practice.
4. Reflect on the group’s learning process: What insights did you gain from working collaboratively?
